# Mobile Diagnostics Based on Motion? A Close Look at Motility Patterns in the Schistosome Life Cycle

**DOI:** 10.3390/diagnostics6020024

**Published:** 2016-06-17

**Authors:** Ewert Linder, Sami Varjo, Cecilia Thors

**Affiliations:** 1 Department of Microbiology, Tumor and Cell Biuology, Karolinska Institutet, SE-17177 Stockholm, Sweden; 2 Center for Machine Vision and Signal Analysis, University of Oulu, FI-90014 Oulu, Finland; samivarj@ee.oulu.fi; 3 Public Health Agency of Sweden, SE-17182 Solna, Sweden; cecilia.thors@folkhalsomyndigheten.se

**Keywords:** POC diagnostics, schistosomiasis, motility patterns, mini-microscopes, image analysis, computer vision, telemedicine, neglected diseases, remote sensing, spectrogram

## Abstract

Imaging at high resolution and subsequent image analysis with modified mobile phones have the potential to solve problems related to microscopy-based diagnostics of parasitic infections in many endemic regions. Diagnostics using the computing power of “smartphones” is not restricted by limited expertise or limitations set by visual perception of a microscopist. Thus diagnostics currently almost exclusively dependent on recognition of morphological features of pathogenic organisms could be based on additional properties, such as motility characteristics recognizable by computer vision. Of special interest are infectious larval stages and “micro swimmers” of e.g., the schistosome life cycle, which infect the intermediate and definitive hosts, respectively. The ciliated miracidium, emerges from the excreted egg upon its contact with water. This means that for diagnostics, recognition of a swimming miracidium is equivalent to recognition of an egg. The motility pattern of miracidia could be defined by computer vision and used as a diagnostic criterion. To develop motility pattern-based diagnostics of schistosomiasis using simple imaging devices, we analyzed *Paramecium* as a model for the schistosome miracidium. As a model for invasive nematodes, such as strongyloids and filaria, we examined a different type of motility in the apathogenic nematode *Turbatrix,* the “vinegar eel.” The results of motion time and frequency analysis suggest that target motility may be expressed as specific spectrograms serving as “diagnostic fingerprints.”

## 1. Introduction

Diagnostics is a vital component of any strategy aiming at control of parasitic infections in endemic regions. Diagnostic dilemmas arise as control measures lead to decreased prevalence and higher demands for sensitivity [1]. Microscopy for the detection of helminth eggs in stools using the Kato Katz method is an efficient tool in high-endemicity situations, but as adequate drug treatments for morbidity control are introduced, the test fails both as a diagnostic tool and as a ‘gold standard’ due to its low sensitivity (~50%) [2,3]. As the demand for cost effectiveness sets limits to available time and other resources, it is usually not possible to increase the sensitivity by increasing the number of test samples, but modifications, such as the use of a specially designed processing chamber, FLOTAC [4], may yield higher sensitivity of microscopy [5]. An additional problem is that diagnostics based on microscopy is difficult to maintain in poor environments, which leads to a spiral of increasing misdiagnosis and neglect [6,7,8,9]. The failure to maintain quality assurance of classical microscopy-based diagnostics is due to a lack of skilled technicians and the failure to maintain equipment usually not designed for prevailing harsh environments [10].

Tests for specific antibodies are useful for the detection of infection in travelers and tourists [11], but in general are of limited importance in endemic regions [12]. Alternative technologies [12] like antigen [13] and nucleic acid detection [14] have so far failed to replace microscopy as they lack the capacity to simultaneously identify a number of possible targets and the information obtained may require interpretation by specialists [15].

The mobile phone has become not only a smartphone computer, but also a microscope as it enters healthcare environments as a diagnostic tool [16,17]. Mini-microscopes with a resolving power approaching that of standard microscopes can be constructed from mobile phone cameras. The limited field of view is a problem that can be solved using wide field on-chip imaging [18,19] or a device for scanning the specimen [20,21]. The fact that images of pathogens can be obtained with a smartphone microscope opens the way for image interpretation by computer vision, which can be performed both locally in the smartphone and at a distance.

Here we explore, as an extension of previous work using computer vision for identification of schistosome eggs based on their morphology [22], the possibility that specific motility characteristics of a parasite could be diagnostic. Diagnostics of several parasite infections are aided by identifying motile organisms. Examples are microfilariae and flagellates in blood or tissues and nematodes in stools. Motility-based diagnostics of schistosomiasis was an established method in China and part of a large-scale—but largely futile—eradication program based on transmission control [23].

The diagnostic hatch test is based on the observation that by adding water to excreted eggs, they will liberate ciliated larvae, which can then be detected by microscopy and even with the naked eye. The hatch test for intestinal schistosomiasis has maintained its role in control programs in China [9,24]. It is performed essentially as described some 50 years ago [25,26,27]. We suggest that the hatch test can be updated and modified by imaging with a smartphone microscope and using computer vision for interpretation.

To further explore motility patterns as potentially diagnostic features, we look at various life stages of schistosomes using available video recordings. Computer vision was used to identify motility in terms of speed, oscillation, contraction frequency, and amplitude. To evaluate our approach from a POC point of view, we used simple mobile phone-based imaging solutions to obtain video recordings of two apathogenic model organisms.

Simple imaging devices, obtained by fitting a lens on top of a mobile phone camera or even by getting rid of the optics and imaging directly on an image sensor, can substitute for conventional microscopes. Connectivity provided by the Internet changes the conditions for health management, disease surveillance [28,29,30,31,32], and interpretation of images obtained with a microscope [16,33,34].

## 2. Materials and Methods

### 2.1. Microscopy and Video Recordings

A Leitz (Leica DMRB) research microscope and an Olympus 3D preparation microscope equipped with a Sony CCD-IRIS video camera model SSC-M370CE were used for video recordings of different schistosome life stages. The frame rate for these video recordings was 15.6 frames per second.

Phase contrast microscopy was performed both for high and moderate magnifications using both microscopes.

Video tape recordings to video home system (VHS) cassettes were originally obtained using a Panasonic videocassette recorder (VCR) Model NV-F65EB. The analog to digital video conversion was originally done using a Sony video editing deck DSR-30P. Recently analog videos were digitized with an Elgato video capture software using the VCR connected via a SCART adapter and a composite video/RCA stereo cable to a MacBook Air computer and imported in H.264 format to iMovie for generation of videos in QuickTime (QT) format.

### 2.2. Mini-Microscopes and Video

High magnification images for video capture were obtained by placing an add-on lens on top of an iPhone 4S mobile phone camera lens. Various alternatives were used: a 4× objective (Olympus), a 35 mm macro lens with iris diaphragm (”Summar” Ernst Leitz GmbH, Wetzlar, Germany), a *Keeploop^®^* macro lens developed at VTT Technical Research Centre of Finland, and Nokia phone camera lenses used in E71 and Lumia smartphones.

An iPhone 4S equipped with a lens from *Keeploop^®^* (02150 Esbo, Finland) (http://www.keeploop.com/, Lens: Fixed Focus lens. Image area width: 2.0–4.0 mm. Area of the image height: 1.5–3.0 mm. Figure zone depth: 0.05–0.1 mm. Resolution: 6–10 microns) was fitted into the screw cap of a plastic bottle and protected by a coverslip from the aqueous sample in the plastic bottle so that objects on the coverslip were in focus. For illumination we used a UV/blue light emitting diode (LED) (See Results). Dark field illumination was achieved by using a torch or LED lamps at an angle roughly perpendicular to the optical axis. On-chip imaging using two simple webcams stripped of their optics and size and resolution markers were as described previously [22].

### 2.3. Target Organisms

*Schistosoma mansoni* organisms of different stages of the life cycle of the parasite were obtained as described previously [35]. The different *S. mansoni* life cycle stages were from experimentally infected mice and *Biomphalaria* snails as described. *Paramecium* organisms and the free-living nematode in the family *Cephalobidae*, *Turbatrix aceti*, vinegar worm or vinegar eel, found in old vinegar or in rotting fruits and vegetables and available for aquarium fish, were from a local shop for aquarium fish and supplies (Södermalms Akvarieaffär, Stockholm, Sweden).

### 2.4. Image Analysis

The workflow contained the following steps: image acquisition, image preprocessing, image segmentation, and processing the segmented targets.

#### 2.4.1. Image Acquisition

Video sequences were obtained from (a) digitized VHS sequences; (b) IPhone 4S; and (c) webcam recordings.

#### 2.4.2. Image Preprocessing for Removal of Background Noise

This was performed by filtering the image data with a Gaussian 3 × 3 low pass filter with standard deviation of 0.75 after gray-scale conversion [36,37].

#### 2.4.3. Image Segmentation Aiming at Creating Candidate Areas for Further Analysis

The spatial segmentation to separate moving objects from still objects was based on temporally adaptive background subtraction and grey value thresholding [38].

The background frame was formed as a median image from frames before and after the processed frame. The frames used for the background were collected four frames before and four frames after the processed frame, with three frames on either side. The background subtracted data were then converted into masks containing the moving objects by keeping the areas where the absolute change was larger than 4 (of 256 gray values). The masks were cleaned using the morphological operations dilate and erode.

#### 2.4.4. Processing the Segmented Targets to Analyze How the Object Behaves over Time

As the whole image is processed to obtain the location of an extracted segment in each frame, this can be time-consuming. To reduce the amount of data to be processed, Kalman filtering was used. By relating measurements of the location of an object to established models, its future location can be predicted [39].

Image analysis of video recordings was performed by Kalman-based tracking, essentially as described for cell motility analysis in phase contrast microscopy [40]. Each extracted object segment was attached to a Kalman filter with a constant velocity motion model having 20 pixel motion noise. To reject segmentation noise, tracks were required to be at least four consecutive frames long.

Track analysis was performed to extract signals that describe typical motility patterns. Individual objects were extracted from the video frames as cropped images for further analysis of speed, acceleration, and number of sharp turns.

The observed “worm wriggling” motion was converted to signal by aligning the extracted images in a common frame. This requires the detection of the ends of the worm in the image patches. Maximally stable extremal regions were used for extracting individual objects in the mini-patches. The displacement of the worm was measured from a line connecting its ends. For simplicity, the change in projection of the worm middle part in a single axis was monitored after rotating the images so that the worm had a fixed length-wise orientation. This creates an oscillating signal that was further characterized with the Fourier transformation. The work flow is similar to what has been used for analysis of human motion [41].

Fourier transformation converts a signal from the time domain to the frequency domain. That is, the signal is decomposed to a collection of sine functions that together estimate the same waveform as the original signal. Each of the superpositioned sine functions has its own frequency and thus one can have a representation of a signal measured over time in frequency space.

The spectrogram reflects the frequency *versus* time plot, with a third dimension indicating the amplitude of the signal visualized by the intensity or color of each point in the plot. A spectrogram, describing how the spectral density of a signal varies with time, was created from 16 frames for short-time Fourier transformation of a signal using the sliding window approach to create spectrograms essentially as described for audio spectrograms [42].

## 3. Results

### 3.1. Motility Patterns of *Schistosoma mansoni* Life Cycle Stages: From Slow Motion to Ultra Rapid

**Figure 1:**
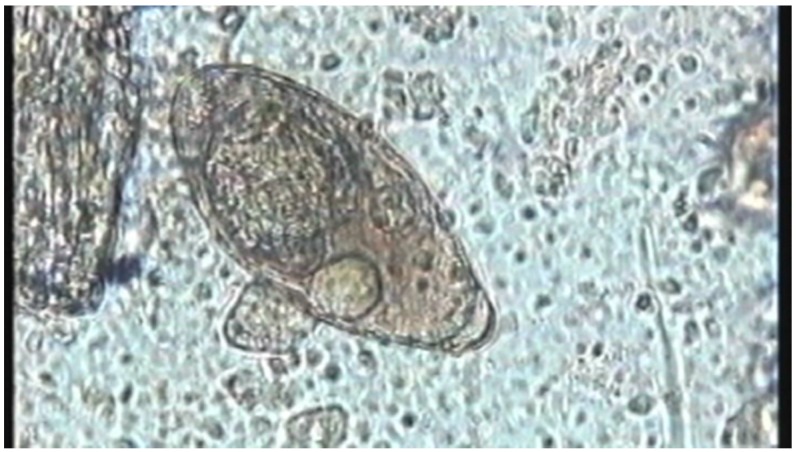
(Video S1) Hatching of *Schistosoma mansoni* egg: Miracidium rotating inside schistosome egg exposed to water. Rotational speed ~14.1 rpm (0.235 rps). Upon bursting of the egg shell (“hatching”), the miracidium rapidly accelerates to achieve a linear speed of about 0.3 mm/s (see Table 1). Digitized VHS-video recording at 15.63 FPS using microscope video camera Sony CCD-IRIS. Original magnification 200×.

**Figure 2:**
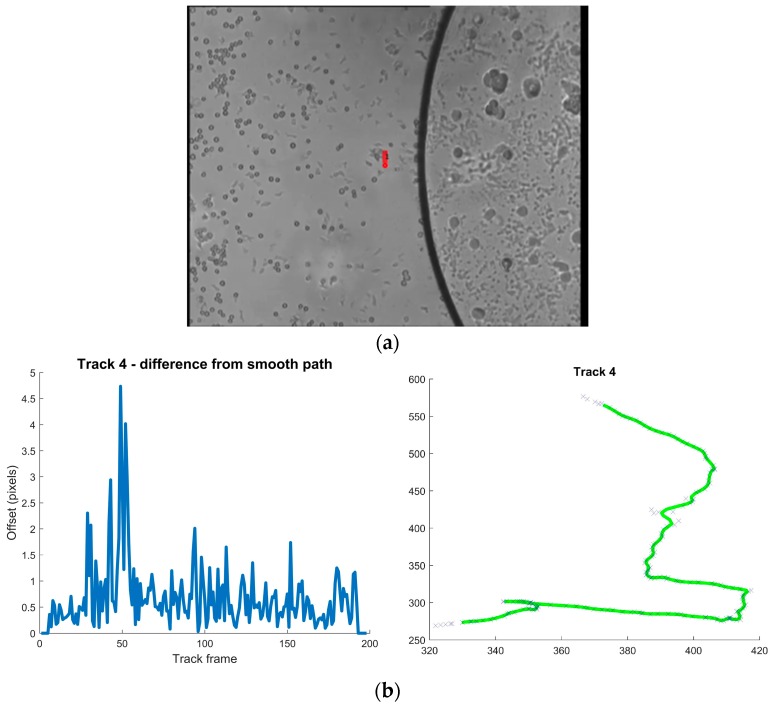
(Video S2) (**a**) Miracidia released from isolated *Schistosoma mansoni* eggs suspended in water swim in droplet on microscope slide under a coverslip. Digitized VHS-video recording as described in the text of Figure 1. Eleven miracidia were identified by computer vision and trajectories of 7 were analyzed in detail (video recording with trajectories). (**b**) The almost linear trajectories of miracidia in water are interrupted as an obstacle is encountered. The graph to the left shows deviations from a straight path and abrupt turnabouts upon contact with an obstacle (an air bubble). The curve to the right (labeled Track 4) shows the rolling average smoothed path in green and measurements corresponding to track 4 seen in Figure 2a.

**Figure 3:**
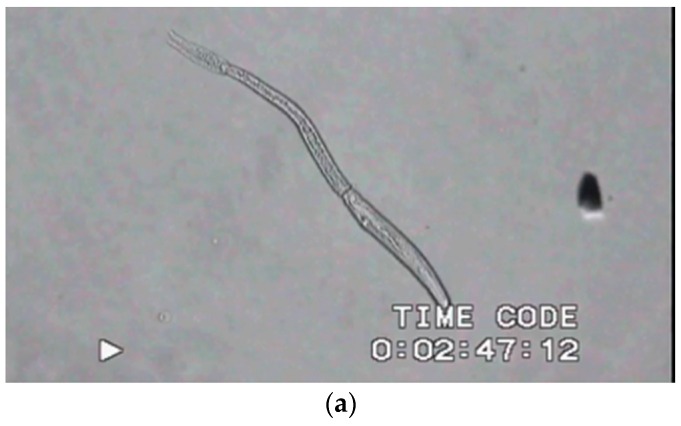

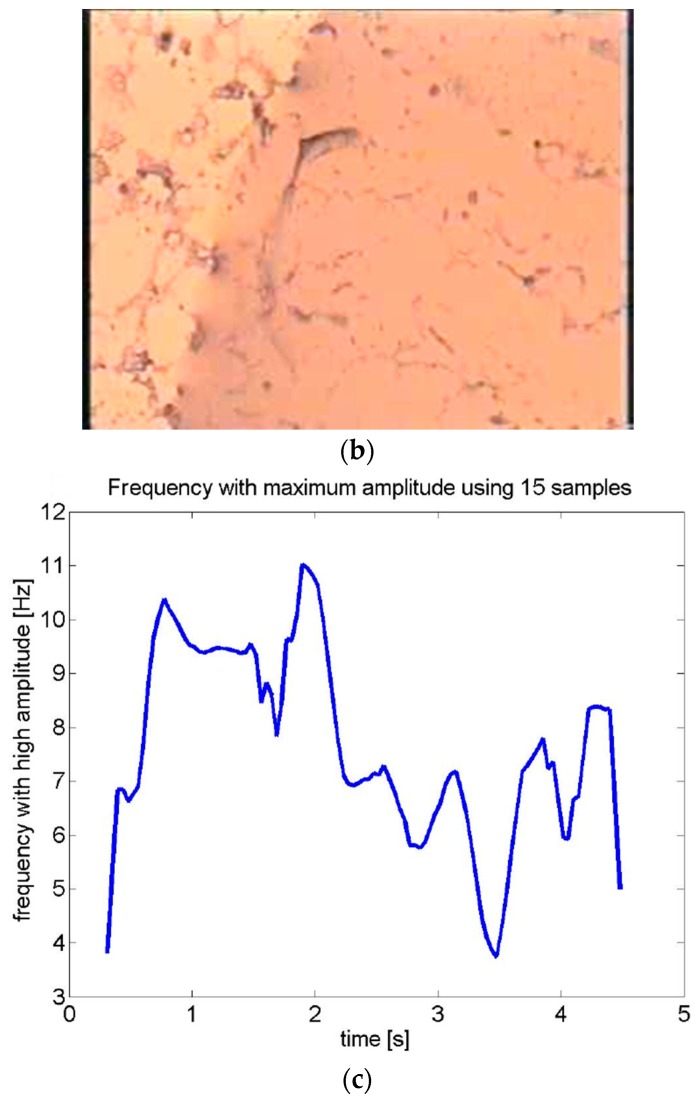
(**a**) (Video S3) *Schistosoma mansoni* cercaria in water. The different motility behavior of the tail and the head parts is seen. The tail will be lost upon penetration of host skin. The frontal part will invade the skin of host and transform into schistosomulum. The recording frame rate 15.63 FPS is too slow to allow for adequate signal processing. (**b**)(Video S4) *Trichobilharzia* cercaria attaches to skin-lipid coated microscope slide and attempts penetration. The wiggling motility of *Trichobilharzia* cercaria was recorded at a frame rate of 15.63 FPS. Original magnification of video recordings was 100×. (**c**) Analysis of cercarial motion seen in **b**; variation of frequency (Hz) over time of 5 s. As it is possible to record a maximum of about 7 or 8 Hz, the recording speed should be at least 2× the frequency maximum. We estimate that to record cercarial motion, at least 100 FPS is needed.

**Figure 4:**
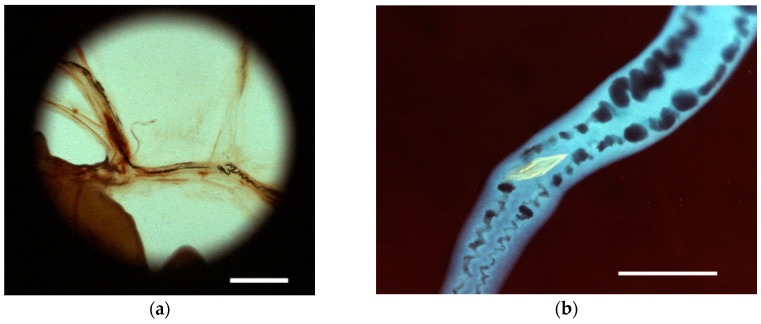

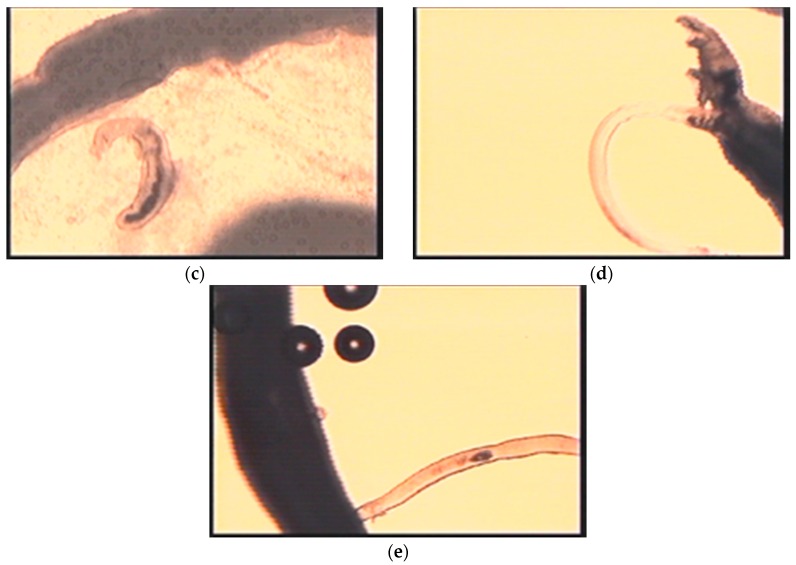
(**a**) Intravascular worms in mesenterial veins of mouse infected with *Schistosoma mansoni*. Note digested erythrocyte pigment present in the intestine of female worms Bar equals 3 mm; (**b**) Intestinal contents and autofluorescent egg in oviduct in isolated female schistosome under UV/blue light Bar equals 200 microns; (**c**) (Video S5) Ingestion of erythrocytes present in the medium; (**d**) (Video S6) Transport of erythrocytes within the gut of female worm; regurgitation within the intestine. In the female worm, egg maturation within the ootype is seen as pulsating, rhythmic contractions at a rate of 156 contractions/min; and (**e**) (Video S7) Egg transportation through the oviduct can be seen as it ends with a conspicuous dorsal nick of the anterior end of the worm as the egg is ejected. Video recordings at 40× magnificatiion at a speed of 15.63 FPS obtained with microscope fitted with 4× objective and video camera Sony CCD-IRIS.

**Figure 5:**
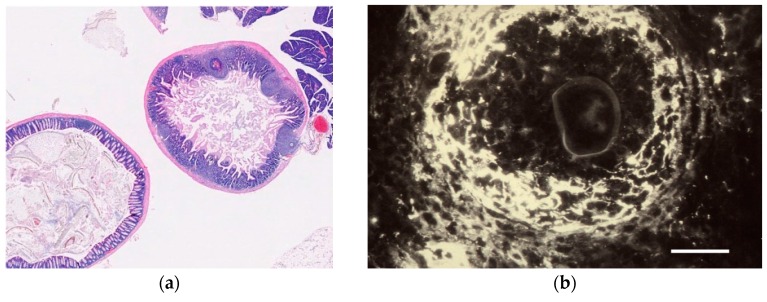
Perioval granulomas in intestinal wall of experimentally infected mouse. (**a**) Paraffin section of intestinal wall stained with hematoxylin and eosin. Virtual microscopy view at 10× magnification: See Webmicroscope for Parasitology [43]. (**b**) Plasmin in periphery of perioval granuloma shown by immunofluorescence. Note slight autofluorescence of eggshell. The perioval host response involves tissue destruction and formation of a tissue channel, in which the egg will be transported through host tissues into the excreta [44]. Bar equals 100 microns.

The VHS video recordings demonstrate several motility patterns reflecting the diverse physiological functions associated with the different life cycle stages of this parasite. (Table 1, Figure 1, Figure 2, Figure 3, Figure 4 and Figure 5).

#### 3.1.1. Eggs and Miracidia

Eggs in contact with water show two types of motility: rotation of the intraoval larva, and its acceleration as the eggshell is bursting or “hatching” (Figure 1). By manual annotation of video frames 1–56, a mean rotational speed of 14.1 rpm was calculated. After bursting of the eggshell, the observed accelerating speed goes from 34 to 455 μm/s—and is still increasing in speed at the last frame (Table 1). In a low-magnification video recording of a water suspension of isolated *S. mansoni* eggs, 11 miracidia were identified and trajectories of seven were analyzed in detail (Figure 2 and Table 2). The swimming patterns are almost linear. Analysis of seven trajectories shows wide variation in speed with a mean value of 225.0 μm/s (Table 3) and change in direction upon contact with an obstacle (Table 4).

#### 3.1.2. Cercaria

Fast tail movements can be seen as cercaria swim in water and when scanning skin lipid-coated surfaces for sites of penetration (Figure 3). The recording speed of the video (15.63 FPS) is insufficient for accurate frequency determination of the movement. The estimated frequency appears to be about 100 Hz, which means that recording speeds need to be about 200 FPS. At (attempted) penetration, the tail is discarded and the anterior part of the body, if it successfully enters the host skin, will penetrate tissues by “inchworm” type of motility as a “schistosomulum” and develop into a female or male worm.

#### 3.1.3. Adult Worms

Adult worms can be seen to move with the aid of an anterior and a posterior sucker *in vitro* in a nutrient solution containing RPMI and red cells. This corresponds to the type of movement worms use when moving in mesenteric vessels (Figure 4a). It is possible to observe motility associated with red cell ingestion and the processing of ingested material within the gut (Figure 4). Female and male schistosomes residing in the mesenteric veins have distinct morphology and motility.

*Schistosoma mansoni* is a parasitic trematode of the portal–mesenteric veins with a closed-end intestine. Adult worms regurgitate their intestinal content after digestion, together with constituents of the lining gut. Figure 4 shows adult worms expressing different mobility patterns. Worm pairs located in mesenteric vessels migrate between the liver and intestine and can be seen based on the large amount of pigment derived from ingested red cells. In the female worm, egg maturation within the ootype is taking place with eggshell formation from components of the surrounding gland cells seen as repeated contractile movements. Egg transportation through the oviduct can be seen as it ends with a conspicuous dorsal nick of the anterior end of the worm.

### 3.2. Videos Captured Using Mini-Microscopes

**Figure 6:**
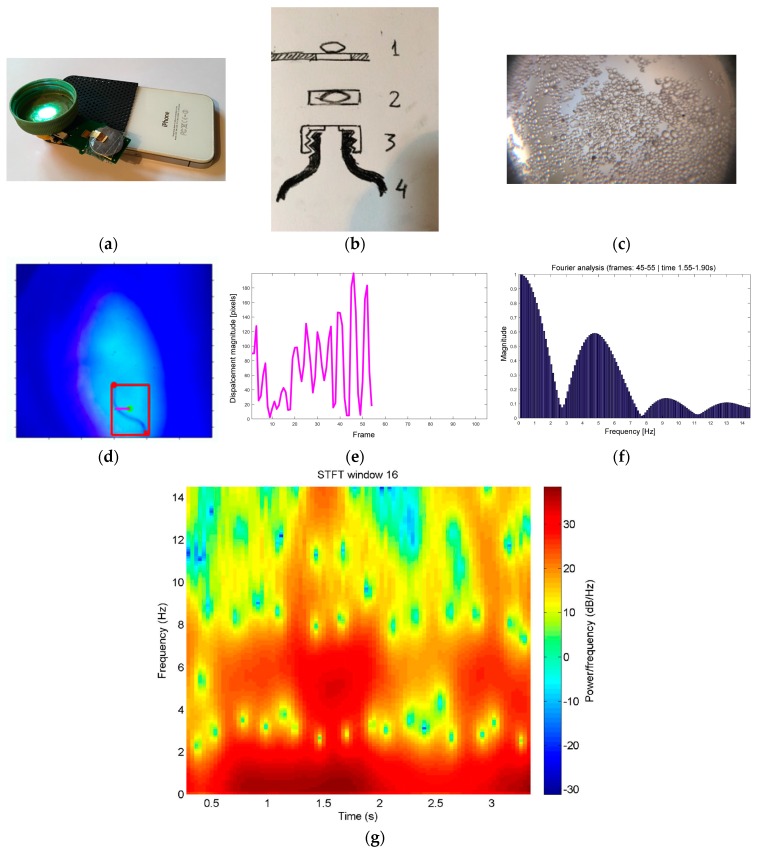
(**a**) The imaging device: Video capture with iPhone 4S at a frame rate of 25 FPS using add-on lens with resolving power of about 10 microns from KeepLoop^®^ fitted into screw cap of plastic bottle containing 10 mL water sample; (**b**) Schematic figure. 1. iPhone 4S; 2. KeepLoop^®^ lens; 3. Screw cap with protective coverslip at focal distance; 4. Plastic bottle containing sample; (**c**) (Video S8) Video recording of marker beads 5–200 microns in diameter suspended in water. Imaging with iPhone 4S and screw cap-mounted lens; (**d**–**f**) (Video S9) *Turbatrix aceti* at the water/air interphase. Illumination using external UV/blue light emitting diode (LED). Computer vision: The worm was extracted using Maximally Stable Extremal region, its ends were located, and the worm displacement from the line connecting the ends was monitored over time. The obtained oscillating signal was analyzed using Fourier transformation. The work flow is similar to what has been used for analysis of human motion [45]; (**g**) Spectrogram of *Turbatrix aceti* motility based on motility signal analysis. The most significant activity occurs at 1.5 seconds, having the fundamental frequency at 5 Hz. While typically used for sound analysis such as singing voice recognition, it is suggested that the spectrogram could be used as a feature source for motion classification [46].

The motility of the *Turbatrix aceti* nematode at the water/air interphase, as captured with an iPhone attached to a PET bottle, is analyzed. The data show that the worm is most active between seconds 1 and 2 of the video and the main frequency is about 5 Hz (Figure 6).

**Figure 7:**
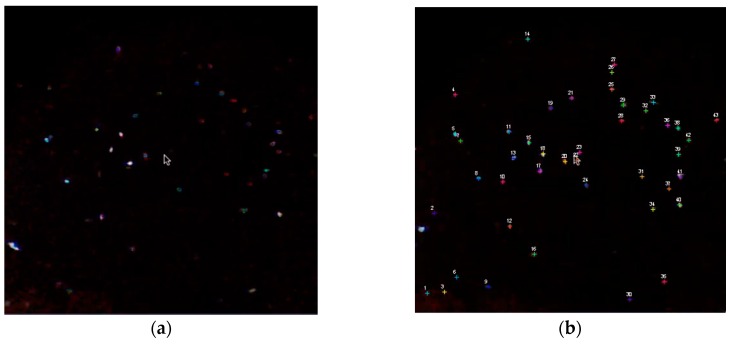
(**a**) (Video S10) Swimming protozoa: *Paramecium* organisms swimming in water were captured with iPhone 4S equipped with 4× objective; 100-mL sample in glass jar. Dark field illumination using flashlight. The dark field imaging facilitates the segmentation of object candidates. (**b**) (Video S11) Individual trajectories of *Paramecium* have a waveform apparently due to the spiral/helical motion of the organism. The waveforms seen apparently represent a helical path and the rotational speed is deductible from the time interval between two maximal readings (see Discussion). Computer vision using analysis of data as described by Kaakinen *et al.* [40].

Swimming protozoa: *Paramecium* organisms swimming in water were captured with an iPhone 4S equipped with 4× objective. We collected a 100-mL sample in a glass jar. Dark field illumination was achieved with a flashlight. Image analysis shows trajectories with a regular wave form (Figure 7).

## 4. Discussion

For identification of targets, typical morphological features need to be recognized. Imaging devices need to generate images containing sufficient information. Depending on the properties of the target organisms, trade-offs are made between magnification, resolution, field of view, focal depth, variations in lighting and image dynamic range, and so on. Identification of excreted schistosome eggs by computer vision is possible and potentially useful [22]. However, identification may not only depend on morphology of target organisms. Here we suggest that motility characteristics, as identified by computer vision, could be diagnostic. The low sensitivity of microscopy as a diagnostic tool and the under-diagnosis of schistosomiasis [2,3] does not reflect difficulties in recognizing the distinct morphology of schistosome eggs. The microscope is an excellent tool, but the problem is that, for several reasons, it is not universally useful. However, microscopy-based diagnostics is not obsolete. Imaging with mini-microscopes and image analysis by computer vision provide tools for signal perception and processing that exceed the capacity of human vision. Our results suggest that motility patterns can be regarded as diagnostic features using unsophisticated microscopes.

The results are discussed from different points of view:

- Motility patterns observed in schistosomes and in two apathogenic species: *Turbatrix*, a nematode, and *Paramecium*, a protozoan.
- Video recording capability of unsophisticated imaging devices and target processing.
- The image analysis process as time and frequency signal processing for identification of motility patterns, “diagnostic fingerprints.”
- Possible target organisms and apathogenic model organisms for a motility analysis-based approach to diagnostics.

### 4.1. Motility Patterns Observed

To identify motility patterns possibly exploitable for diagnostic purposes as mobile microscopes, we examined VHS video recordings using low power phase contrast microscopy of *S. mansoni* life cycle stages. While the motility of miracidia could be characterized from the low frame rate recordings (15.63 FPS), these videos could not accurately determine the frequency of cercarial movements. Their estimated frequency appears to be about 100 Hz, which means that the recording speed needs to be about 200 FPS, which is possible with smartphones now on the market. VHS video recordings were used in our study for two reasons: they were available based on previously unpublished work, and new experiments could not be performed since the schistosome life cycle had been discontinued. The fact that miracidial trajectories could be analyzed based on low frame rate videos is of practical importance considering mobile phone-based imaging devices for POC diagnosis. Future experiments to this end should benefit from our results using *Turbatrix* and *Paramecium* as apathogenic model organisms.

#### 4.1.1. Schistosomes

As summarized in Table 1, the life cycle of schistosomes includes the development of parasite stages in both a definitive mammalian host and an intermediate snail host, which expresses various types of motility. To maintain the life cycle, this highly successful parasite [47] has developed distinct motile stages for invasion of both definitive and intermediate hosts. The cercaria emerging from infected snails swim with the aid of a bifurcated tail, which is lost upon host invasion. The invading anterior part, the schistosomulum, migrates through tissues and matures into male or female adult worms that end up mating in the blood stream, apparently attracted to each other [48,49] by TGF-β pathway signaling [50]. *S. haematobium*, the causative agent for urinary schistosomiasis, becomes resident in the vasculature surrounding the bladder, where the female parasite deposits eggs that end up in the urine. Adult worms of *S. mansoni*, *S. japonicum*, and other related species reside in the mesenteric and hepatic veins and the eggs produced end up in stools. Excreted eggs transported into the environment release their larval content, the miracidium, upon contact with water.

Miracidia remain alive for several hours and swim by means of surface cilia to find a distinct snail species for invasion. At penetration of the snail, the surface ciliary coat is lost and the miracidium undergoes extensive multiplication and transformation to generate infectious cercaria via a sporocyst stage.

The two larval stages, the cercaria and the miracidia, show distinct motility behaviors, depending on muscular contractions and ciliary beating, respectively.

There is evidence for the female worms being capable of entering the terminal branches of the mesenteric veins to deposit their eggs into tissues surrounding the gut and bladder, respectively. *In vitro* observations of egg production gives a clue to the mechanism for egg deposition, dorsoflexion of the anterior part of the female as the egg is ejected. The leakage of intraoval parasite components—identified as glycoconjugates by lectin affinity [51,52]—into the surrounding tissue leads to a vigorous host response, seen as a granuloma surrounding the eggs in tissues (http://www.webmicroscope.net/parasitology/). This inflammatory response involving plasminogen activation creates a channel through which the egg is transported through the sub-epithelial and epithelial tissues into the excretions.

In addition to these major expressions of motility, it is possible to observe motility in association with eggshell formation and transport of the eggs inside the oviduct. Also, the ciliary movements in protonephridial elements can be seen in both miracidia and adult worms.

#### 4.1.2. Turbatrix and Paramecium

Motility of these two species was quite distinct, but remarkably similar to motility patterns observed in stages of the schistosome life cycle. The difference reflects the physiological difference between motility based on muscular contractions and ciliary movement. The wiggling motility patterns of *Turbartrix aceti* and schistosome cercaria were similar and could be expressed in terms of frequency and amplitude (see below). The swimming pattern of schistosome miracidia resembled that of *Paramecium*, although the trajectories of the two organisms were distinct. The miracidia moved in an almost linear path with a mean speed of 225.0 μm/s, whereas *Paramecium* trajectories had a wavy shape, likely to reflect a spiral motion [53] similar to the motility patterns described for sperm and other 3D microswimmers [54,55]. The waveforms seen apparently represent a helical path and the rotational speed is deductible from the time interval between two maximal readings. Interpretation of the wave forms as 2D representations of 3D motion give us the rotational speeds. For track 25, four waves are seen in 6 s, which corresponds to 0.67 rps *i.e.*, 40 rpm. Track 16: ~9/6 s ≥ 90 rpm, track 5:10/9 s ≥ 66 rpm, track 36: 5/5 s ≥ 60rpm, T22 ~10/11 ≥ 54rpm.

### 4.2. Image Capture and Video Recording

Digitized VHS recordings could be analyzed by computer vision even if the rather poor resolution—maximum of 250–300 × 480 pixels—and frame rate of about 15 FPS of the original videos set limits to the information that could be extracted. However, they show numerous motility patterns that could be analyzed in detail by computer vision. The information on the larval stages, both the cercarial and miracidial motilities, can be regarded as the basis for more detailed studies related to diagnostics and the analysis of water quality in the future.

The video recording capability of the iPhone 4S in combination with rather simple optics allowed for rather sophisticated computer vision analysis.

Analysis of miracidia trajectories based on higher quality video recordings is directly linked to the possibility of developing a diagnostic hatching test for schistosomiasis based on computer vision, as discussed below.

We did not try to optimize illumination, resolution, contrast, or field of view. A major imaging problem encountered is due to the fact that live target organisms have a density similar to that of the surrounding aqueous medium. This problem was approached by using dark field illumination or oblique light.

Preliminary experiments using on-chip imaging with modified webcams for video recording of *Paramecium* as described previously [53] were not successful, apparently because turbulence caused by heat from the sensor chip surface interfered with movement and gave distorted trajectories. Therefore add-on magnifying lenses of various kinds were used. The video recordings of *Turbatrix aceti* were obtained with a macro lens from a commercially available “pocket microscope“, *Keeploop*^®^. We originally thought that the illumination through the specially designed lens, which is generated by two LEDs, would provide light for a dark-field illumination. Increasing the amount of light had only a limited effect on the contrast between the object and the background. Useful images of the nematode were, however, obtained at the air–water interface, where the contrast was sufficient. These observations motivate development of optimal dark field illumination designed for objects with an optical density similar to water. Thus, optimized microfluidic solutions are required for specimen handling and visualization [22,54,56].

Imaging devices for POC diagnostics have to meet different requirements—technical, economic, social, and cultural. Technical requirements such as resolution and field of view depend not only on properties of target organisms but also on factors such as type of sample and the concentration of targets.

Target organisms differ in properties such as size, morphological features, and optical density. They may also have distinguishing properties that can be exploited for diagnostics, such as birefringence [57,58], autofluorescence [59,60], or magnetic [61], adhesive, antigenic, and lectin-binding properties [62,63].

Depending on the requirements and circumstances, imaging can be done using a number of innovative mobile microscope solutions [16]. Add-on lenses of different kinds including ball-lenses, mobile camera lenses [21,64,65], and lens arrays have been used [66,67,68], as well as lens-free on-chip imaging devices [18,32,54,69,70]. Sophisticated optical and illumination systems and lens-free on-chip holographic imaging with image reconstruction can achieve high-resolution images, wide field of view, and enhanced contrast comparable to—or even surpassing—the image quality of conventional microscopes [16,54,69,71,72,73].

### 4.3. Image Analysis

To recognize motion is a relatively simple task and for many diagnostic applications a simple “burglar alarm” type of application may be sufficient. A number of smartphone apps may be useful e.g., for recognition of Strongyloides worms in stools. Numerous tracking algorithms have been published [74] and new ones like LOCOMOTIS [75] allow for inexpensive tracking analysis. More advanced signal processing was achieved through the approach of Kaakinen *et al*. [40]. The degree of sophistication required obviously depends on what type of information is required. The image analysis process we used aimed at highly specific target identification based on motility time and frequency signal processing. Observed motility patterns might then serve as “diagnostic fingerprints.” Our results suggest that this is achievable.

Identification of motility by computer vision is a major component not only in weapons technology and robotics, but also in traffic and home security. In these areas signal processing—of different complexity—is of central importance. However, the basic ideas are not new and signal processing is a mature field. Fourier analysis to understand the information contents of a signal was proposed about 70 years ago [76].

The segmentation methods are not always very specific; it is typical to have segments that clearly do not represent the objects of interest, and these have to be removed from the detections. This is a classification task (also called pattern recognition) where the classification method can range from simple object area-based rejection to deep neural networks, representing the state of the art in machine learning [77].

Classification is often based on features that are extracted from the segmented area. The features are then compared to a model for decision-making. Simplifying the machine learning and artificial intelligence research is all about how to learn and use good models efficiently [78].

Our results suggest that motility-based identification of miracidia is possible by computer vision. The deep learning approach to pattern recognition and machine learning has recently attained a lot of attention due to the success in many classification tasks. The principle is that features are not hand crafted but are learned from training data. The learned neural network structure can present features at different complexity levels, finally leading to decision classes [79]. Many factors determine which classification method to select for a specific task. It depends on factors such as size of available training data and the difficulty of the task. While simple classifiers can be created with hundreds of training samples, large multiclass classifiers based on deep networks may use millions of training samples.

Signal amplification is an emerging concept with unexpected consequences and an apparent potential also in the diagnostic field and in video image interpretation of the diverse motility patterns described here [80].

### 4.4. Motility-Based Diagnostics—Obvious and Not-So-Obvious Applications

Current diagnostics of several parasite infections is based on finding motile organisms in patient samples. Microfilaria in blood, Strongyloides larvae in feces, trypanosomes, Leishmania, and other flagellates such as Trichomonas are examples, and it is logical to develop computer-assisted detection methods based on target motility for diagnosing infections caused by these organisms. In fact, detection of microfilaria motility may be sufficient for identification against a background of immobile blood cells [21,64], trypanosomes and other flagellates, malaria gametocytes, and trematode cercariae.

Motility-based diagnostics, which characterizes movement in terms of speed, frequency, and type of motion, may be developed based on current methods used for drug testing on hookworm [81] and other helminths such as *Ostertagia ostertagi*, *Cooperia oncophora*, and *Haemonchus contortus* [82].

The wiggling motion of *Turbatrix* at the air–water interface recorded with mobile phone and add-on lens was up to 14 Hz, which is the maximum frequency that can be recorded with the standard video recording speed of 24 FPS achieved with the iPhone 4S. While we here demonstrate the principle, additional data are required to study the robustness of the approach by determining both the lengths of video recordings and the frame rates needed for recording a certain type of motility. It is clear from the information theory that the frame rate should be at least twice the highest frequency that we want to observe. To study cercarial motion, novel smartphones (not used in the present study) can be used as they can record video at frame rates 10 times higher than those used in the present study. These devices also take time-lapse recordings useful for studies of slowly moving objects like amoebas. As shown for speed measurements of miracidia in this study, the variation needs to be determined, as it is likely that individual and strain differences, specimen handling, age, different matrix, *etc.* affect the swimming behavior.

The hatch test [25] takes advantage of the phototactic behavior of *S. mansoni* miracidia to attract the organism to the region of the hatching chamber where inspection can be done under optimal illumination. Whereas phase contrast illumination is the standard in studies of live cells in culture, dark field illumination appears to be useful for visualizing organisms in water, and the data have a more suitable background for several machine vision algorithms.

The varying success of the schistosome egg hatching technique [24,26] suggests that standardized procedures and further evaluation of sensitivity and specificity are needed considering the difficulties posed by the absence of an adequate “gold standard” [2,83]. Taking advantage of the possibilities provided by mini-microscopes and computer vision, it may be possible to increase the performance of diagnostics by combining target identification based on both morphology and motility.

Observations by Upatham, Sturrock, and others that *S. mansoni* eggs will remain viable in the stool for about a week if they are not subjected to heat or drying and that miracidia remain motile for 8–12 h are of considerable practical importance [84,85].

The maximum observed speed of miracidia in water was about 0.5 mm/s, as calculated from videos of hatching eggs obtained at two separate occasions from experimentally infected mice. This is not very different from the speed reported previously (2 mm/s) [86,87]. Both methodological and biological dissimilarities may account for the observed difference.

Clearly the standard VHS recordings of cercarial motility fail to give an accurate frequency of the tail motion due to this limitation in recording speed. An estimated frequency of the cercarial motion is 50–100 Hz, which means that newer mobile phone “slow motion” video recordings at speeds of 200–1000 FPS should suffice for accurate determination.

The observed straight linear swimming behavior of miracidia appears to be distinct from the rotating, spiral, or helical motion of *Paramecium* shown previously [53], and, interestingly, also to that of trypanosomes [88], sperm, and *Giardia* [73]. Whether the slight wave shape of some parts of miracidial trajectories (e.g., track 11 in Figure 2) represents helical movement remains to be determined by using 3D imaging [56], e.g., as described for *Giardia* [73]. Apparently the overall direction of a spiral trajectory moving in water is maintained to compensate for asymmetries in body shape in a similar way as a rotating bullet leaving a gun barrel maintains its linear path. Further experiments on hatching of helminth eggs are required to determine whether linear trajectories and the capacity of miracidia for rapid change of direction are unique to *S. mansoni*.

Taken together, our results suggest that it is possible to consider motion frequency and amplitude, shape of trajectories, linear and rotational speed as putative diagnostic features, in a similar way as morphological features are diagnostic in current microscopy. The summary information obtained by computer vision can be expressed as spectrograms, which may serve as a “diagnostic fingerprint.”

Recording of motility patterns for diagnostics could be employed for diverse purposes in addition to diagnostics based on egg hatching, e.g., water analysis for presence of cercaria (risk for swimmer’s itch and schistosomiasis). Peculiar motility patterns, like those seen in egg maturation and transport in the intravascular female schistosome, may be diagnostic using future techniques such as photoacoustic microscopy and wireless endoscopic imaging in combination with motion amplification and image analysis [80,89]. Motility-based diagnostics could be used for identification of a variety of motile helminths and protozoa, but also to analyze specimens after induction of motile stages, like after excystation of protozoan cysts and after induction of flagellated gametocytes in patient blood samples to study malaria transmission [90].

### 4.5. Model Organisms and Future Studies

To develop both hardware and software solutions for motility diagnostics, controlled experiments obviously need to be performed—while considering the possible difference between *in vitro* and *in vivo* conditions [88]. Apathogenic model organisms [91] are necessary as prototype imaging devices containing microfluidic chambers, filtration barriers, lighting conditions, *etc.* need to be tested and evaluated under defined and secure conditions. Experiments using the two organisms *Turbartrix* and *Paramecium* illustrate this point and also demonstrate that no advanced culture facilities are needed for their maintenance. Both organisms used in these experiments were easily available as they are sold as aquarium fish food. *Turbatrix aceti* and *Caenorhabditis elegans* are useful apathogenic nematodes, which can be used as models for pathogens like Filaria and Strongyloides. Paramecium can be used as model organisms for schistosome miracidia as shown in this study. Several model organisms are offered as biology kits for science education (see e.g., [92]).

The motility analysis performed in our study suggests that schistosome miracidia can be identified based on their motility using computer vision. Whether this diagnostic approach will be useful as an alternative to improved morphology-based identification remains to be tested. The protocol for motility-based diagnostics of intestinal schistosomiasis would be simple: Place stool sample in water to induce hatching, and look for miracidia by computer vision to identify typical trajectories. Diagnostics of urinary schistosomiasis seems to be a logical starting point for validation, as excreted *S. haematobium* eggs can be isolated using simple filtration techniques, e.g., as described recently [93] and hatching then induced by suspension of eggs into water. *S. mansoni* miracidia could be concentrated at the field of view of the camera, taking advantage of the phototactic property of these organisms [25]. Spectrograms obtained as the result of image analysis can be considered fingerprints of the movement profile in much the same way that speech spectrograms can be used to identify speakers [42].

## 5. Conclusions

Recognition of moving organisms facilitates microscopy-based diagnostics of a number of parasites. We suggest that motility can be exploited further as a diagnostic feature by imaging devices such as smartphone-based mini-microscopes and tools for signal analysis provided by computer vision. This approach would bring about characterization of motility in a detailed, exact, and reproducible way, in terms of speed, acceleration, amplitude, frequency, *etc.* Video recordings of different life stages of schistosomes and of two model organisms were analyzed to explore the diagnostic potential of motility parameters as seen by computer vision. Our results suggest that motility can be defined based on “diagnostic fingerprints” obtained by signal processing. Such signals could be obtained from video recordings at modest resolutions and frame rates. Our results suggest that mobile microscopes and computer vision can be used to detect schistosomiasis based on the motility pattern of miracidia. By exploiting their combined potential, motility patterns could serve as additional diagnostic markers in infections currently diagnosed by microscopy.

## Figures and Tables

**Table 1:** Observed motility in the different life stages of *Schistosoma mansoni* (The life cycle was maintained in mice as definitive hosts and *Biomphalaria glabrata* intermediate hosts as described in [35]).

Environment	Location in Host	Stage	Purpose of Motility	Type of Motility	Reference
Definitive Host/Human, Mammalian	Tissue	Schistosomulum	Migration to mesenteric vessels	Inchworm	-
	Blood	Adult worms	Ingestion of RBCs	Contractions	Videos; Figure 4a,c,d
			Metabolism	Peristaltics	
			Mating	Attraction	-
			Excretory function of protonephridia	Ciliary beating	-
		Adult female only	Egg maturation and shell formation	Ootype contractions	Videos; Figure 4d
			Egg ejection/Deposition in tissues	Transport/Dorsoflexion/Expulsion	Videos; Figure 4c
		Adult male only	Sperm	(?)	-
	Tissue	Eggs	Egg migration into excreta	Granuloma formation	Videos; Figure 5
Water		Eggs	Hatching/Bursting of egg shell	Rotation of intraoval miracidium	Video S1
		Miracidia	Finding intermediate host	Swimming	Video S1; Video 2
Intermediate host/Snail	Tissue	Sporocyst	-	-	-
Water		Cercaria	Host finding	Swimming	-
			Attachment/Penetration into host	Wiggling/Tail loss/	Video S3; Figure 3b
				Inchworm	-

**Table 2:** Acceleration (mm/s^2^) of “hatched” miracidium seen in Figure 2.

Frame	Time (s)	Velocity (mm/s)	Acceleration (mm/s^2^)
75	2.503	0.076	2.269
76	2.536	0.034	−1.253
78	2.569	0.104	2.104
79	2.603	0.076	−0.851
80	2.636	0.096	0.620
81	2.669	0.069	−0.830
82	2.703	0.217	4.439
83	2.736	0.058	−4.774
84	2.769	0.295	7.130
85	2.803	0.349	1.594
86	2.836	0.455	3.189

**Table 3:** Motility trajectories of *Schistosoma mansoni* miracidia after egg hatching in water Analysis of video recording (Figure 2) by image analysis software [40].

Trajectory ID	Mean Velocity (μm/s)	Track Length (mm)
1	123.6	10.49
2	329.0	3.77
3	152.6	12.60
4	261.2	12.60
5	213.0	12.60
6	139.3	12.60
11	356.0	5.12

Mean velocity of six miracidial trajectories: 225.0 μm/s calculated based on an estimated size of 100 μm of miracidia, corresponding to 15.9 pixels and frame rate of 15.63 FPS.

**Table 4:** Analysis of six *Schistosoma mansoni* miracidia trajectories in video recording (see Figure 2).

Track	Mean Difference from Smoothed Path Pixels	Max Pixels	Direction Changes (mean) Degrees/ms	Max Degrees/ms
1	0.464	1.623	0.407	2.814
3	0.428	1.543	0.199	1.044
4	0.664	4.737	0.177	1.066
5	0.601	5.547	0.210	1.027
6	0.355	2.060	0.176	1.041
11	0.887	7.668	0.091	0.428

## Supplementary Materials

Supplementary File 1

Supplementary File 2

Supplementary File 3

Supplementary File 4

Supplementary File 5

Supplementary File 6

Supplementary File 7

Supplementary File 8

Supplementary File 9

Supplementary File 10

Supplementary File 11

## Abbreviations

The following abbreviations are used in this manuscript:

POC	Point of Care
LED	Light-Emitting Diode
VHS	Video Home System
FPS	Frames per Second
KI	Karolinska Institutet, Stockholm, Sweden
FIMM	Institute for Molecular Medicine Finland
